# Association of Admission Medication Regimen Complexity with Delirium in Critically Ill Adults: A Prospective Multicenter Cohort Study

**DOI:** 10.3390/jcm15218457

**Published:** 2026-10-30

**Authors:** Abrar Alotaibi, Maram Alshreef, Qoot Almedainy, Alaa Almagthali, Ahmed Basilim, Ahmad Majrashi, Abdulhamid Althagafi, Eman Alqahtani, Qasem Bahri, Dina Kutbi, Arwa Albeladi

**Affiliations:** 1 Department of Clinical Pharmacy, Al Hada Armed Forces Hospital, Taif 26792, Saudi Arabia; 2 Department of Clinical Pharmacy, King Abdulaziz University Hospital, Jeddah 21589, Saudi Arabia; 3 Department of Pharmaceutical Services, Prince Sultan Military Medical City, Riyadh 12233, Saudi Arabia; 4 Department of Pharmacy Practice, Faculty of Pharmacy, King Abdulaziz University, Jeddah 22254, Saudi Arabia; abasilim@kau.edu.sa (A.B.);; 5 Department of Clinical Pharmacy, King Fahad Central Hospital, Jazan 82666, Saudi Arabia; 6 Department of Pharmacy, King Fahad Armed Forces Hospital, Jeddah 23311, Saudi Arabia; 7 College of Pharmacy, Batterjee Medical College, Jeddah 21442, Saudi Arabia

**Keywords:** intensive care unit, delirium, medication regimen complexity, mortality, length of stay

## Abstract

**Background:** The medication regimen complexity–intensive care unit (MRC-ICU) score is a validated tool for assessing medication regimen complexity in critically ill patients and has been associated with several clinical outcomes. However, its association with delirium remains poorly characterized. This study aimed to evaluate the association between the day-1 MRC-ICU score and delirium development and to describe associated clinical outcomes. **Methods:** This prospective multicenter cohort study enrolled 364 adult patients admitted to intensive care units (ICUs) across four tertiary hospitals between January and April 2025. MRC-ICU, sequential organ failure assessment, and acute physiology and chronic health evaluation II (APACHE II) scores were recorded within 24 h of admission. The primary outcome was delirium during the ICU stay. Secondary outcomes were acute kidney injury (AKI), fluid overload (FO), renal replacement therapy (RRT), ICU and hospital mortality, and ICU and hospital length of stay. Logistic regression was used to assess the association between day-1 MRC-ICU score and delirium. **Results:** Delirium occurred in 12 patients (3.3%). Baseline MRC-ICU scores did not significantly differ between the delirium and no-delirium groups (median, 12 vs. 11; *p* = 0.364). No statistically significant association was detected per 5-point increase in the day-1 MRC-ICU score in either the unadjusted model (odds ratio [OR] 1.28, 95% confidence interval [CI] 0.73–2.23; *p* = 0.385) or the APACHE II-adjusted model (OR 1.27, 95% CI 0.73–2.22; *p* = 0.398). Patients who developed delirium had higher rates of incident AKI (33.3% vs. 4.5%; *p* = 0.009), FO (83.3% vs. 10.5%), RRT initiation (66.7% vs. 4.3%), ICU mortality (83.3% vs. 12.5%), and hospital mortality (100% vs. 17.0%) (*p* < 0.001 for the remaining outcomes). **Conclusions:** No statistically significant association was detected between the day-1 MRC-ICU score and delirium development. Delirium was associated with higher rates of adverse clinical outcomes. However, the small number of delirium events and imprecise estimates preclude definitive conclusions regarding the association between admission medication regimen complexity and delirium risk.

## 1. Introduction

Delirium, a frequent and clinically important manifestation of acute neurologic dysfunction in critically ill patients, is associated with prolonged hospitalization, increased mortality, greater resource utilization, and long-term impairment in cognitive and functional recovery among patients admitted to the intensive care unit (ICU) [1,2]. Delirium develops within a highly dynamic environment characterized by organ failure, invasive support, sedation, infection, and rapid therapeutic changes; therefore, identifying practical markers that summarize treatment intensity and medication-related burden at the bedside is of great interest.

Critically ill patients commonly receive multiple high-risk medications, including sedatives, analgesics, vasopressors, antimicrobials, anticoagulants, electrolyte replacements, gastrointestinal prophylaxis, and rescue medications. Medication count cannot fully capture this complexity, because medications differ in route, monitoring burden, titration intensity, and clinical risk. Numerous studies on the complexity of medication regimens have long documented that regimen burden is multidimensional rather than merely numerical. A systematic review describing approaches to measure medication regimen complexity emphasized the need for context-specific tools that reflect real-world clinical demands rather than raw polypharmacy alone [3].

To address this gap in critical care, the medication regimen complexity-ICU score (MRC-ICU) was developed as a scoring tool specifically for critically ill patients in the ICU [4]. The original study has established a 39-item instrument intended to more accurately reflect the complexity of ICU pharmacotherapy than medication count alone. A follow-up multicenter validation study demonstrated convergent and divergent validity across medical and surgical ICU settings [5]. A modified version of the MRC-ICU score was proposed to simplify assessment while preserving its clinical signal [6], positioning it as a more clinically meaningful representation of ICU medication burden than total medication count alone by incorporating the types of medications used and the intensity of ICU-specific pharmacotherapy.

Studies on the MRC-ICU score expand beyond score development, with reports linking higher MRC-ICU scores to inpatient mortality, longer ICU and hospital length of stay, fluid overload (FO), drug–drug interactions, and pharmacist interventions [7,8,9,10,11,12,13]. These findings indicate that medication regimen complexity reflects a clinically relevant dimension of ICU care. Concurrently, several studies have emphasized the operational value of the MRC-ICU score for pharmacist workload assessment and service prioritization [9,10,11,12,14,15], reinforced by reports describing successful automation and electronic health record embedding of the MRC-ICU score calculation, enabling real-time use in clinical workflows without requiring manual scoring for every patient [12,16].

Even with these advances, whether the MRC-ICU score can help identify neurologic complications, such as delirium, remains unclear. Delirium is not only a marker of disease severity but also arises from interactions among patient vulnerability, acute organ dysfunction, inflammation, sedation and analgesia strategies, sleep disruption, immobility, infection, and medication exposure. Given that medication management is deeply intertwined with each of these domains, an ICU-specific medication complexity score is conceptually relevant to delirium risk, with a higher complexity score indicating greater exposure to treatable agents, more severe instability, broader rescue therapy, and heavier pharmacologic management. However, it is also possible that regimen complexity is more strongly aligned with general treatment intensity and pharmacist workload than with the specific pathobiology or clinical diagnosis of delirium.

This distinction is important in both research and practice. If medication regimen complexity is associated with delirium, the MRC-ICU score can be utilized as an additional risk-stratification tool, potentially supporting targeted surveillance and preventive interventions. Conversely, the absence of such an association is equally informative by suggesting that baseline global medication complexity should not be treated as a surrogate for vulnerability to delirium. Studies to date have primarily evaluated mortality, length of stay, fluid balance, pharmacist interventions, and implementation feasibility [7,8,9,10,11,12,13,16,17], whereas the relationship between the MRC-ICU score and delirium is insufficiently characterized.

Therefore, we conducted a prospective multicenter study to examine whether the MRC-ICU score, determined on day 1 of ICU admission, was associated with the subsequent development of delirium in critically ill adult patients. Additionally, we compared important clinical outcomes, including acute kidney injury (AKI), FO, renal replacement therapy (RRT), mortality, and length of stay, among patients stratified by delirium status.

## 2. Materials and Methods

### 2.1. Study Design and Setting

This prospective multicenter cohort study across four tertiary hospitals enrolled adult patients admitted to the medical or surgical ICU for at least 72 h between January and April 2025. Both delirium and FO were prespecified primary outcomes in the original study protocol. The present analysis focuses on delirium, while the exclusion criteria related to renal replacement therapy and high-output fluid losses were established partly to support the assessment of FO. Patients with delirium present at ICU admission were excluded to ensure that the primary outcome represented new-onset delirium during the ICU stay. Patients with end-stage renal disease, defined as an estimated glomerular filtration rate < 15 mL/min/1.73 m^2^, those receiving chronic maintenance RRT or acute RRT at ICU admission, those with clinician-documented FO at ICU admission, and those with diagnoses associated with substantial fluid loss, including high-output fistulas (>500 mL/day) or high-output drains (>500 mL/day), were excluded, as these conditions could confound fluid balance assessment and potentially influence the study outcomes. Clinician documentation was used to identify FO at ICU admission because reliable cumulative fluid balance data before ICU admission were unavailable.

These fluid-related exclusion criteria also supported the assessment of FO as another prespecified primary outcome. Pre-existing cognitive impairment, dementia, alcohol withdrawal, neurological injuries, and communication barriers were not specified as additional exclusion criteria. Eligibility criteria were applied by the clinical pharmacists during prospective patient enrollment. Only patients meeting the inclusion criteria and none of the exclusion criteria were included in the study dataset. Patients who did not meet the eligibility criteria were not recorded, and a screening log documenting the total number of patients assessed for eligibility and the reasons for exclusion was not maintained. Consequently, these numbers could not be reliably reconstructed. The completed Strengthening the Reporting of Observational Studies in Epidemiology (STROBE) checklist is provided in Supplementary Table S1.

### 2.2. Data Collection

Clinical and demographic data were prospectively collected using a standardized data collection form. The collected variables included age, sex, body weight, height, and body mass index as well as comorbidities, such as diabetes mellitus, hypertension, coronary artery disease, chronic kidney disease, chronic obstructive pulmonary disease, and heart failure. The primary ICU diagnosis was categorized as neurologic, pulmonary, cardiovascular, infectious, gastrointestinal, renal, endocrine, dermatologic, trauma, and neoplastic conditions. Disease severity was assessed using the sequential organ failure assessment (SOFA) and acute physiology and chronic health evaluation II (APACHE II) scores determined within the first 24 h of ICU admission. Mechanical ventilation status at ICU admission was also recorded. 

Medication regimen complexity was assessed using the original 39-item MRC-ICU scoring tool across all four participating hospitals, based on medications actually administered during the first 24 h of ICU admission, to evaluate its association with subsequent delirium. MRC-ICU scores were calculated by trained clinical pharmacists using standardized scoring instructions across all four participating hospitals. MRC-ICU scores were not collected after this period.

### 2.3. Study Outcome

The primary outcome was the development of delirium during ICU admission. Delirium was identified through clinical pharmacist review of documented Confusion Assessment Method for the Intensive Care Unit (CAM-ICU) assessments and clinical notes in the medical record. A positive CAM-ICU assessment or a clinician-documented diagnosis of delirium was considered evidence of delirium. The clinical teams routinely performed delirium screening across all participating hospitals, generally twice daily; clinical pharmacists reviewed the records to ascertain the outcome. Delirium motor subtypes, including hypoactive, hyperactive, and mixed delirium, were not separately classified. Data on Richmond Agitation–Sedation Scale (RASS) scores and the assessability of deeply sedated or comatose patients were not systematically collected. Therefore, the number of patients who were unassessable for delirium could not be determined. 

AKI was defined according to the Kidney Disease: Improving Global Outcomes (KDIGO) criteria, based on changes in serum creatinine and urine output, using the serum creatinine level at ICU admission as the baseline reference. Incident FO was defined as a cumulative positive fluid balance of ≥10% of the patient’s body weight at ICU admission or a new clinician-documented diagnosis of FO during the ICU stay. Meeting either criterion was sufficient for classification. Cumulative fluid balance was assessed from ICU admission because reliable pre-ICU fluid balance data were unavailable. RRT was initiated at the discretion of the treating clinical team based on standard clinical indications, including refractory hyperkalemia, severe metabolic acidosis, FO, or uremic complications.

Secondary outcomes included incident AKI during ICU stay, incident FO during ICU admission, initiation of RRT, ICU mortality, hospital mortality, ICU length of stay, and hospital length of stay. Incident outcomes were defined as events occurring during ICU admission among patients who did not have the condition at ICU admission. For the analysis of incident AKI, patients with AKI already present at ICU admission were excluded from the population at risk.

### 2.4. Ethical Considerations

The study protocol was approved by the Research Ethics Committees of the participating institutions (Institutional review board no: 139-25, E-2743, REC 862, and 2566). The requirement for informed consent was waived due to the observational study design and the use of routinely collected clinical data. Delirium assessments were performed by the treating clinical teams as part of routine ICU care, while the research team reviewed documented CAM-ICU assessments and clinical records without conducting additional patient assessments or interventions specifically for research purposes.

### 2.5. Statistical Analysis

Continuous variables were presented as medians with interquartile ranges (IQRs), whereas categorical variables were expressed as numbers and percentages. Comparisons between patients who developed delirium and those who did not were performed using the Mann–Whitney *U* test for continuous variables and the chi-square or Fisher’s exact test for categorical variables, as appropriate. Logistic regression analysis was performed to evaluate the association between the baseline MRC-ICU score on day 1 and the development of delirium. An adjusted model including the APACHE II score was used to account for disease severity. Multicollinearity between admission MRC-ICU and APACHE II scores was assessed using the variance inflation factor (VIF). Given the limited number of delirium events, the primary adjusted logistic regression model was restricted to these two predictors to reduce the risk of overfitting. Missing data were handled using complete-case analysis, without imputation. 

Patients with missing APACHE II scores were excluded from the adjusted logistic regression model, whereas all patients with available day-1 MRC-ICU scores and delirium outcomes were eligible for the unadjusted analysis. Results were reported as odds ratios with 95% confidence intervals. The association between MRC-ICU score and delirium was additionally expressed as an odds ratio per 5-point increase in the day-1 MRC-ICU score. The discriminative ability of the day-1 MRC-ICU score for delirium was evaluated using receiver operating characteristic (ROC) curve analysis, with the area under the curve (AUC) and its 95% confidence interval estimated using 12,000 stratified bootstrap resamples. Given the small number of delirium events, Firth’s penalized logistic regression was performed as a sensitivity analysis to reduce small-sample bias. Penalized profile likelihood confidence intervals and likelihood ratio tests were used for inference. An additional exploratory Firth penalized logistic regression model including age and neurological admission diagnosis, alongside the day-1 MRC-ICU and APACHE II scores, was performed to assess the sensitivity of the findings to adjustment for potential confounding. Statistical analyses were performed using SPSS version 29 (IBM, Armonk, NY, USA), with additional ROC bootstrap and Firth penalized logistic regression analyses conducted using Python version 3.12.14. A two-sided *p*-value < 0.05 was considered statistically significant.

## 3. Results

**Figure 1:**
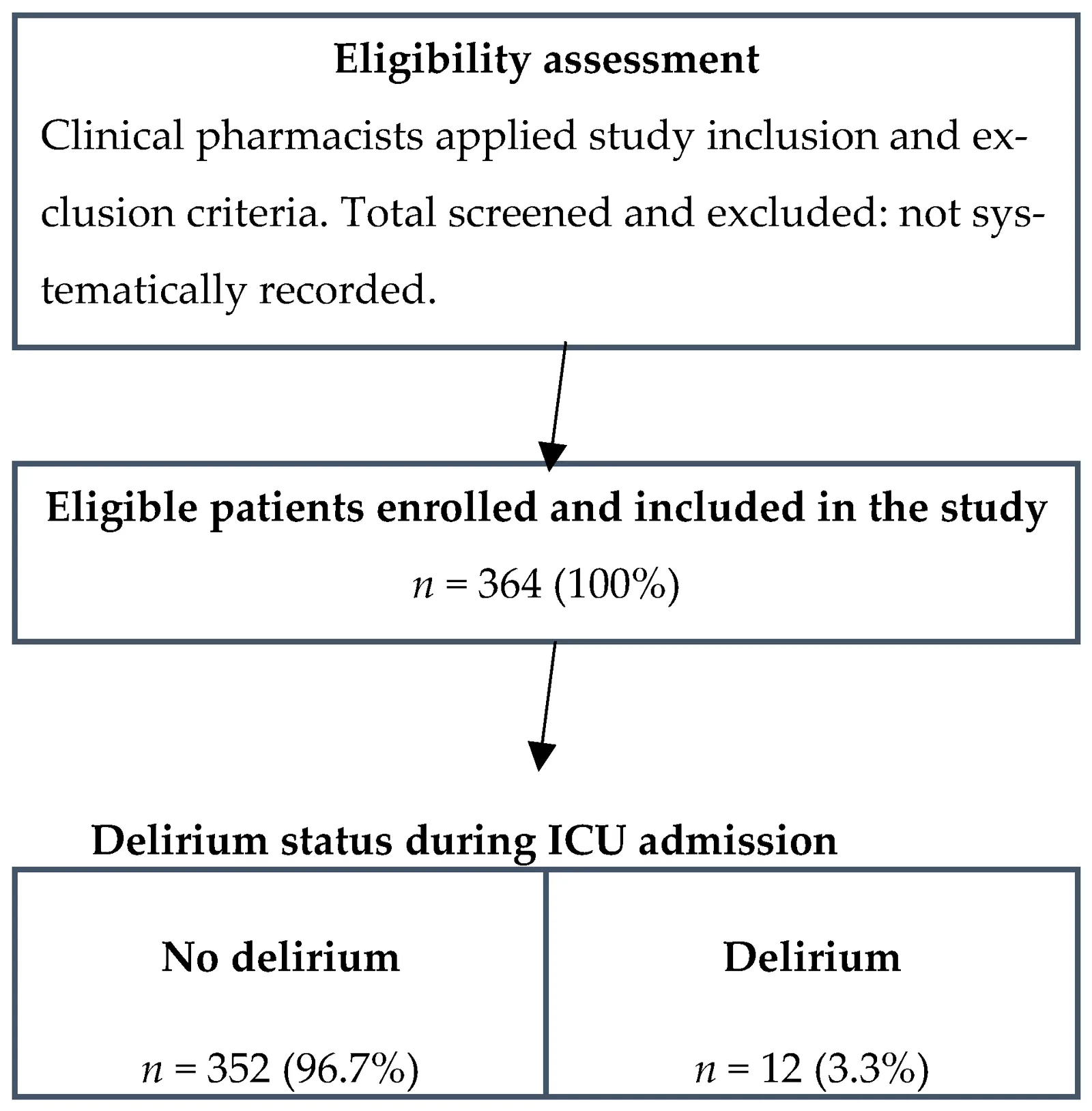
Flow diagram of study participants according to delirium status. Figure legend. Eligibility criteria were applied by clinical pharmacists during prospective enrollment. The total numbers screened and excluded, including reasons for exclusion, were not systematically recorded. All 364 enrolled patients were included in the study; 12 developed delirium during ICU stay and 352 did not.

The study cohort comprised 364 critically ill patients, including 12 (3.3%) who developed delirium during their ICU stay (Figure 1). Table 1 summarizes the baseline demographic and clinical characteristics of patients with and without delirium. Briefly, patients with delirium were significantly younger than those without delirium (median age, 39.5 [IQR 29.3–56] vs. 61 [IQR 40–74] years; *p* = 0.012). However, other characteristics, including weight, height, and body mass index, did not significantly differ between the two groups.

Disease severity at ICU admission, as assessed by the SOFA and APACHE II scores, was comparable between patients with and without delirium (median SOFA score, 7 [IQR 4.8–8.3] and 6 [IQR 4–8], respectively; *p* = 0.570; median APACHE II score, 14 [IQR 10.8–18] and 16 [IQR 9–21], respectively; *p* = 0.541). Similarly, the baseline MRC-ICU score on day 1 of ICU admission did not significantly differ between patients with and without delirium (median, 12 [IQR 9.8–18] and 11 [IQR 8.8–15], respectively; *p* = 0.364).

Regarding baseline clinical characteristics, the proportion of patients with neurologic diseases was significantly higher in patients with delirium than in those without delirium (50.0% vs. 23.3%; *p* = 0.044), whereas the prevalence rates of other baseline comorbidities, including diabetes mellitus, hypertension, chronic kidney disease, coronary artery disease, heart failure, chronic obstructive pulmonary disease, and dyslipidemia, were not significantly different between the two groups.

Unadjusted comparisons of clinical outcomes according to delirium status are presented in Table 2. Patients who developed delirium had higher observed rates of renal complications than those who did not develop delirium. Among patients without AKI at ICU admission, incident AKI occurred in 3 of 9 patients (33.3%) who developed delirium, compared with 11 of 246 patients (4.5%) without delirium (*p* = 0.009). FO was also more frequent among patients who developed delirium (83.3% vs. 10.5%; *p* < 0.001), as was RRT initiation during ICU stay (66.7% vs. 4.3%; *p* < 0.001). These comparisons were unadjusted, and the temporal sequence between delirium and these outcomes could not be established.

Notably, mortality outcomes were significantly higher in patients with delirium than in those without delirium. Specifically, ICU mortality was significantly higher in patients with delirium than in those without delirium (83.3% vs. 12.5%; *p* < 0.001), whereas hospital mortality was nearly universal in those with delirium (100% vs. 17.0%; *p* < 0.001). Additionally, delirium was associated with prolonged stay in the ICU and the hospital (median ICU length of stay, 28 [IQR 14–45] vs. 6 [IQR 4–16] days; *p* < 0.001; median hospital length of stay, 56 [IQR 32–78] vs. 18 [IQR 10–34] days; *p* < 0.001).

Logistic regression analysis was performed to evaluate the association of the MRC-ICU score measured on day 1 of ICU admission and the development of delirium. As summarized in Table 3, no statistically significant association was detected per 5-point increase in the MRC-ICU score on day 1 in either the unadjusted model (OR 1.05, 95% CI 0.94–1.17; *p* = 0.385) or in the model adjusted for the APACHE II score (OR 1.05, 95% CI 0.94–1.17; *p* = 0.398). However, the confidence intervals were sufficiently wide that a clinically meaningful association could not be excluded. Multicollinearity assessment showed a VIF of 1.01 for both admission MRC-ICU and APACHE II scores, indicating no evidence of problematic multicollinearity between the predictors included in the adjusted model. APACHE II scores were missing for 17 patients (4.7%). Consequently, the adjusted logistic regression model included 347 patients, including all 12 patients who developed delirium. In the adjusted model, the APACHE II score was not significantly associated with delirium (OR 0.99 per 1-point increase, 95% CI 0.93–1.05; *p* = 0.705).

ROC analysis of the day-1 MRC-ICU score yielded an AUC of 0.577 (95% bootstrap CI 0.401–0.746), indicating modest discrimination with considerable uncertainty. Sensitivity analysis using Firth’s penalized logistic regression yielded findings consistent with the conventional logistic regression models. For each 5-point increase in the day-1 MRC-ICU score, the unadjusted OR was 1.29 (95% CI 0.73–2.21; *p* = 0.367), while the APACHE II-adjusted OR was 1.30 (95% CI 0.74–2.22; *p* = 0.360). These findings remained statistically nonsignificant, although clinically meaningful associations could not be excluded. In an additional exploratory Firth regression model adjusting for APACHE II score, age, and neurological admission diagnosis, the association between the day-1 MRC-ICU score and delirium remained statistically nonsignificant (OR 1.16 per 5-point increase, 95% CI 0.65–2.04; *p* = 0.604). Given the limited number of delirium events, these findings should be interpreted cautiously.

## 4. Discussion

In this prospective multicenter cohort of 364 critically ill adults, no statistically significant association was detected between day-1 medication regimen complexity, measured using the MRC-ICU score, and subsequent delirium development. Nevertheless, the limited number of delirium events and the width of the confidence intervals indicate substantial uncertainty, and a clinically meaningful association cannot be excluded. Conversely, patients who developed delirium experienced substantially worse clinical outcomes, including markedly higher AKI rates, FO, RRT, ICU mortality, hospital mortality, and longer ICU and hospital length of stay.

The present study has two primary findings. First, the day-1 MRC-ICU score did not demonstrate a statistically significant association with subsequent delirium development in this cohort, although the small number of events limits the certainty of this finding. Second, delirium identified a subgroup of patients with pronounced adverse outcomes across renal complications, healthcare resource utilization, and mortality. These observations are clinically relevant because they highlight the adverse clinical outcomes observed among patients with delirium, while the association between the day-1 MRC-ICU score and subsequent delirium remains uncertain given the small number of delirium events.

Our findings should be interpreted within the context of published studies on the MRC-ICU score. Since its introduction and validation, the MRC-ICU score has been shown to correlate with medication count, patient acuity, mortality, ICU length of stay, FO, pharmacist interventions, and drug–drug interactions [4,5,6,7,8,9,10,11,12,13]. Multicenter studies have strengthened the external validity of this tool, and implementation studies based on electronic health records have supported its operational use in routine care [5,12,16]. However, published applications of the MRC-ICU score have primarily focused on mortality prediction, workload triage, and other ICU outcomes rather than delirium. Therefore, the present study addresses an outcome that has not been a standard target of prior investigations on the MRC-ICU score.

The current results also align with an important conceptual feature of the MRC-ICU score: This tool is a global measure of medication regimen complexity, not a delirium-specific pharmacologic index, a likely important distinction. The MRC-ICU score was designed to summarize ICU medication burden and complexity in a meaningful way for critical care practice [4,5,6]; thus, it may perform best for questions related to global treatment intensity, resource utilization, pharmacist activity, or broad clinical outcomes that closely align with overall complexity [7,8,9,10,11,12,14]. In contrast, delirium may be driven by a more selective subset of medications and by nonpharmacologic factors that are only partially represented within a global medication complexity score.

Established ICU delirium prediction models, including PRE-DELIRIC and E-PRE-DELIRIC, incorporate multiple clinical predictors rather than relying on medication regimen complexity alone [18,19]. Unlike these delirium-specific models, the MRC-ICU score was developed to quantify overall medication regimen complexity rather than the neurocognitive effects of individual medications. Previous research has identified sedatives, particularly benzodiazepines, and medications with anticholinergic properties as potentially important contributors to delirium risk [20,21]. These differences highlight the need to distinguish overall medication regimen complexity from medication-specific delirium risk. Future studies should investigate whether incorporating MRC-ICU scores alongside established delirium predictors and measures of deliriogenic medication exposure improves delirium risk assessment.

There are several potential explanations for the absence of a significant association between the day-1 MRC-ICU score and delirium in the current cohort. Delirium is multifactorial and may not be adequately captured by a single baseline summary of medication complexity alone [22]. The MRC-ICU score was designed to capture overall regimen burden in the ICU, not specifically the neurocognitive impact of sedative, anticholinergic, or psychoactive medication exposure. A patient may have a high medication complexity score because of vasopressors, antibiotics, electrolyte replacements, or prophylactic therapies, without necessarily carrying a corresponding increase in delirium risk. Conversely, a patient may develop delirium because of risk factors that are incompletely reflected by the baseline medication regimen complexity, such as underlying vulnerability, evolving organ dysfunction, sedation strategy over time, infection-related encephalopathy, sleep disruption, or cumulative treatment exposure after the first day of ICU stay. The timing of exposure assessment may also matter. Several studies on the MRC-ICU score have demonstrated that medication regimen complexity changes over time and that repeated measures can capture dynamic differences over the course of the ICU stay [6,12]. Likewise, delirium is a time-dependent outcome. Therefore, a one-time MRC-ICU score on day 1 may provide an incomplete representation of the medication-related environment that precedes the onset of delirium. ICU management involves multiple evolving therapeutic interventions, including vasoactive support, analgesia, sedation, antimicrobial therapy, renal support, and fluid management, as reflected in contemporary critical care guidelines [23,24]. The lack of an association observed in the present study does not preclude the possibility that operationalizing other aspects of medication regimen complexity, such as peak complexity, cumulative burden, and time-varying trajectories, may yield different results.

Although no statistically significant association was detected between the day-1 MRC-ICU score and delirium, the clinical profile of patients who developed delirium is noteworthy. Compared with patients without delirium, those with delirium were younger and more frequently had neurologic diagnoses at baseline. The younger age of patients who developed delirium should be interpreted cautiously, given that only 12 delirium events occurred. Although neurologic admission diagnoses were more frequent in this group, specific diagnoses were unavailable for further characterization, and the reason for the age difference remains uncertain. This finding should not be interpreted as evidence that younger age independently increases delirium risk. Although the primary logistic regression model adjusted for APACHE II, which incorporates age, an additional exploratory Firth penalized regression model included age and neurological admission diagnosis as separate covariates. The association between the day-1 MRC-ICU score and delirium remained statistically nonsignificant in this sensitivity analysis. Nevertheless, the small number of delirium events and potential underdiagnosis of delirium prevent definitive conclusions regarding the observed age difference. Disease severity at admission, measured by the SOFA and APACHE II scores, did not significantly differ between the two groups, and day-1 MRC-ICU scores were also comparable. Patients who developed delirium also had higher observed rates of clinical complications and greater healthcare resource utilization. These findings may reflect concurrent manifestations of underlying illness severity and physiological deterioration rather than a causal relationship.

Previous research has demonstrated an association between the MRC-ICU score and FO in critically ill patients [13]. In the present study, patients who developed delirium had higher observed rates of AKI, FO, and RRT initiation. These findings may reflect concurrent manifestations of underlying physiological deterioration and the need for organ support rather than a causal relationship between delirium and renal complications.

Mortality outcomes were significantly different between the patients with and without delirium in the present cohort. Prior studies on the MRC-ICU score observed associations between higher medication regimen complexity and mortality [7,8,9,25]. However, the MRC-ICU score was not significantly associated with delirium in the present study, and delirium was strongly associated with mortality, suggesting that delirium might reflect overall clinical deterioration rather than admission medication complexity in this cohort.

Delirium was also associated with significantly longer ICU and hospital stays, wherein patients with delirium stayed in the ICU nearly five times longer and remained in the hospital more than three times longer than those without delirium. Previous studies on the MRC-ICU score have demonstrated the association of higher regimen complexity with longer length of stay [4,6,7,8,9,10]. However, the observed association between delirium and length of stay should be interpreted cautiously because longer ICU stays provide greater opportunities for delirium detection. Furthermore, the temporal sequence between delirium onset and prolonged hospitalization was not established. Therefore, these findings cannot determine whether delirium contributed to prolonged hospitalization or was more frequently identified among patients with longer and more complicated ICU courses.

Our findings provide insufficient evidence to establish the predictive value of the day-1 MRC-ICU score alone for delirium risk, although the score may still be useful for other purposes described in prior studies, such as describing medication burden, estimating pharmacist workload, and evaluating certain ICU outcomes [7,8,9,10,11,12,13,16]. In contrast, its association with delirium was unclear in our analysis, suggesting that delirium risk assessment may need to incorporate additional factors, including disease severity, neurologic diagnosis, mechanical ventilation, sedation exposure, and other clinical changes over time, rather than depending solely on baseline medication complexity.

These findings may also help define how the MRC-ICU score is used in future ICU research. Prior studies have shown its utility as a medication summary measure in the ICU for observational work, pharmacist workload assessment, and use within electronic health record systems [9,10,11,12,14,16]. However, the predictive performance of the day-1 MRC-ICU score as a standalone marker for delirium risk remains uncertain given the limited number of delirium events. Future studies with larger samples and sufficient delirium events should investigate whether adding repeated MRC-ICU measurements to established clinical prediction models improves their discrimination, calibration, or clinical utility. Such incremental predictive value was not established in the present study.

The present study has several notable strengths. This prospective study included patients from four tertiary care hospitals and focused on delirium, an outcome that has not been well studied in relation to the MRC-ICU score. Baseline variables were collected using a standardized form, and the analyses also included clinically meaningful patient outcomes, including mortality and length of stay. 

We acknowledge several limitations of this study. First, delirium occurred in only 12 patients (3.3%), limiting statistical power and the precision of the regression estimates. No formal a priori sample size calculation was performed specifically for delirium. Therefore, the absence of a statistically significant association should not be interpreted as evidence of no association, as a clinically meaningful association may have been missed. Given the limited number of events, the primary adjusted model included only MRC-ICU and APACHE II scores to reduce overfitting. Although exploratory Firth penalized regression analyses were performed to address small-sample bias and assess potential confounding by age and neurological admission diagnosis, residual confounding and imprecise estimates remain important concerns.

Second, MRC-ICU was assessed only during the first 24 h of ICU admission, preventing evaluation of changes in medication complexity, peak complexity, or cumulative medication burden. Furthermore, individual medication exposures were unavailable, precluding adjustment for potentially deliriogenic medications, including benzodiazepines, opioids, and anticholinergics. Consequently, the admission MRC-ICU score may not adequately represent medication-related delirium risk throughout ICU stay.

Third, delirium ascertainment relied on routinely documented CAM-ICU assessments and clinical diagnoses rather than independent research assessments, potentially resulting in underdiagnosis, particularly of hypoactive delirium. RASS scores, periods of coma or deep sedation, and the timing of delirium assessment relative to death were not systematically recorded. Consequently, the number of unassessable patients or deaths before the first assessment could not be determined, and competing-risk or assessability-restricted analyses were not feasible. Differences in observation time, as well as unmeasured institutional variations in sedation practices, staffing, and documentation, may also have influenced delirium detection.

Fourth, the exclusion of patients with end-stage renal disease, maintenance renal replacement therapy, or high-output fistulas or drains restricted the clinical spectrum of the cohort. Recruitment was also limited to four tertiary hospitals in Saudi Arabia over four months (January–April 2025), potentially limiting generalizability. During prospective enrollment, clinical pharmacists recorded only patients who met the eligibility criteria as part of their routine clinical workflow across the participating hospitals. A separate screening log documenting all patients assessed for eligibility and the reasons for exclusion was not maintained. Consequently, the total numbers of screened and excluded patients could not be reliably reconstructed, limiting the assessment of potential selection bias.

Finally, the observational design and unavailable timing of individual clinical events preclude causal interpretation of the associations between delirium and renal complications, FO, mortality, or length of stay. These findings should therefore be considered exploratory and require confirmation in larger prospective studies.

Overall, the present study contributes new evidence regarding the relationship between medication regimen complexity and delirium in critically ill adults. No statistically significant association was detected between the day-1 MRC-ICU score and delirium, although the limited number of events prevents definitive conclusions. Patients who developed delirium had higher observed rates of adverse clinical outcomes, highlighting the need for further research into delirium and its associated clinical complications in critically ill patients.

## 5. Conclusions

In this prospective multicenter cohort, no statistically significant association was detected between the day-1 MRC-ICU score and delirium development, either before or after adjustment for the APACHE II score. Patients who developed delirium experienced higher rates of AKI, FO, RRT, ICU and hospital mortality, and longer ICU and hospital stays. However, the small number of delirium events limits the precision of the estimates, and a clinically meaningful association between admission MRC-ICU score and delirium cannot be excluded. Further studies with larger samples and more delirium events are needed to clarify this relationship.

## Figures and Tables

**Table 1:** Baseline Demographic and Clinical Characteristics According to Delirium Status.

Variable	Total (*n* = 364)	No Delirium (*n* = 352)	Delirium (*n* = 12)	*p*-Value
Baseline Characteristics				
Age, years	61 (39.8–73)	61 (40–74)	39.5 (29.3–56)	0.012
Weight, kg	70 (60–84)	70 (60–84)	67 (59.5–76.5)	0.645
Height, cm	160 (156–170)	160 (156–170)	160 (155.8–164)	0.523
BMI, kg/m^2^	26.4 (22.5–31.0)	26.4 (22.3–31.0)	26.2 (23.7–31.2)	0.753
Male sex, *n* (%)	167 (45.9%)	160 (45.5%)	7 (58.3%)	0.396
Smoking, *n* (%)	18 (4.9%)	17 (4.8%)	1 (8.3%)	0.461
Mechanical ventilation at admission, *n* (%)	219 (60.2%)	210 (59.7%)	9 (75.0%)	0.376
Assessment of Disease Severity				
SOFA score	6 (4–8)	6 (4–8)	7 (4.8–8.3)	0.570
APACHE II score	16 (9–21)	16 (9–21)	14 (10.8–18)	0.541
MRC-ICU score day 1	11 (9–15)	11 (8.8–15)	12 (9.8–18)	0.364
ICU Type				
Surgical ICU	66 (18.1%)	65 (18.5%)	1 (8.3%)	0.702
Comorbidities, *n* (%)				
Diabetes mellitus	173 (47.5%)	170 (48.3%)	3 (25.0%)	0.145
Hypertension	191 (52.5%)	187 (53.1%)	4 (33.3%)	0.242
CKD	54 (14.8%)	53 (15.1%)	1 (8.3%)	1.000
CAD	35 (9.6%)	35 (9.9%)	0	0.615
Heart failure	22 (6.0%)	22 (6.3%)	0	1.000
COPD	12 (3.3%)	12 (3.4%)	0	1.000
Dyslipidemia	64 (17.6%)	63 (17.9%)	1 (8.3%)	0.700
Admission diagnosis, *n* (%)				
Infection/sepsis	100 (27.5%)	97 (27.6%)	3 (25.0%)	1.000
Neurologic disease	88 (24.2%)	82 (23.3%)	6 (50.0%)	0.044
Cardiovascular disease	65 (17.9%)	64 (18.2%)	1 (8.3%)	0.701
Pulmonary disease	51 (14.0%)	49 (13.9%)	2 (16.7%)	0.679

Data are presented as medians (interquartile range) or *n* (%). Comparisons are performed using the Mann–Whitney U test for continuous variables and the chi-square or Fisher’s exact test for categorical variables. APACHE II, Acute Physiology and Chronic Health Evaluation II; BMI, body mass index; CAD, coronary artery disease; CKD, chronic kidney disease COPD, chronic obstructive pulmonary disease; ICU, intensive care unit; MRC-ICU, Medication Regimen Complexity- Intensive Care Unit; SOFA, Sequential Organ Failure Assessment.

**Table 2:** Clinical Outcomes According to Delirium Status.

Outcome	No Delirium (*n* = 352)	Delirium (*n* = 12)	*p*-Value
Incident AKI during the ICU stay	11/246 (4.5%)	3/9 (33.3%)	0.009 ^a^
Incident fluid overload during the ICU stay	37/352 (10.5%)	10/12 (83.3%)	<0.001 ^a^
Incident RRT during the ICU stay	15/352 (4.3%)	8/12 (66.7%)	<0.001 ^a^
ICU mortality	44/352 (12.5%)	10/12 (83.3%)	<0.001 ^a^
Hospital mortality	60/352 (17.0%)	12/12 (100%)	<0.001 ^a^
ICU length of stay, days	6 (4–16)	28 (14–45)	<0.001 ^b^
Hospital length of stay, days	18 (10–34)	56 (32–78)	<0.001 ^b^

Data are presented as medians (interquartile ranges) or *n* (%). AKI, acute kidney injury; ICU, intensive care unit; RRT, renal replacement therapy. Data represent unadjusted comparisons according to delirium status. Patients receiving RRT or presenting with clinician-documented fluid overload at ICU admission were excluded from the study cohort. For incident AKI, patients with AKI at ICU admission were excluded from the corresponding analysis; the at-risk denominators were 246 patients without delirium and 9 patients with delirium. Hospital length of stay was calculated for all patients, including those who died during hospitalization. The temporal sequence between delirium and other clinical outcomes was not established. ^a^ Fisher’s exact test; ^b^ Mann–Whitney U test.

**Table 3:** Association Between MRC-ICU Score and Delirium.

Variable	OR	95% CI	*p*-Value
MRC-ICU score on day 1 (per 5-point increase, unadjusted; *n* = 364)	1.28	0.73–2.23	0.385
MRC-ICU score on day 1 (per 5-point increase, adjusted for APACHE II; *n* = 347)	1.27	0.73–2.22	0.398
APACHE II score (per 1-point increase, adjusted model; *n* = 347)	0.99	0.93–1.05	0.705

APACHE II, Acute Physiology and Chronic Health Evaluation II; CI, confidence interval; ICU, intensive care unit; MRC-ICU, Medication Regimen Complexity–Intensive Care Unit; OR, odds ratio. The unadjusted analysis included 364 patients, whereas the APACHE II-adjusted analysis included 347 patients because 17 patients had missing APACHE II scores. Missing data were handled using complete-case analysis.

## Data Availability

Data are available upon request from the corresponding author.

## Supplementary Materials

The following supporting information can be downloaded at: https://www.mdpi.com/article/10.3390/jcm15218457/s1, Table S1: STROBE Checklist.
